# Supplementary material for: Lactobacillus plantarum Alters Gut Microbiota and Metabolites Composition to Improve High Starch Metabolism in Megalobrama amblycephala
Source: Animals (Basel). 2025 Feb 18;15(4):583. doi: 10.3390/ani15040583 (PMC11852042; doi:10.3390/ani15040583)
Supplement: Supplementary file 1 [file animals-15-00583-s001.zip › animals-3422958-supplementary.pdf]

Supplementary Data

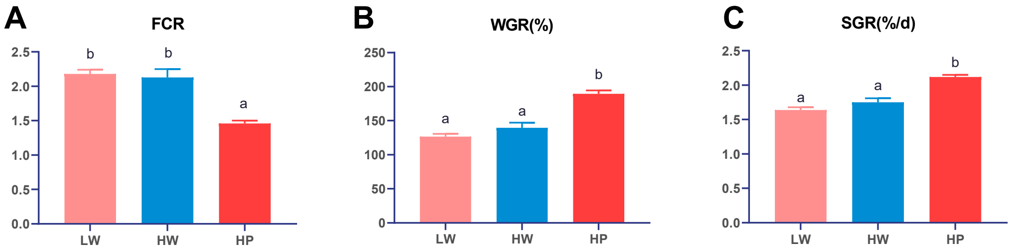

Figure S1. Effects of different experimental diets on growth performance of juvenile *M.* amblycephala. Note: The values in the figure are the mean ± standard error. The different lowercase letters indicated significant differences among groups (*P*<0.05, Turkey's Test).

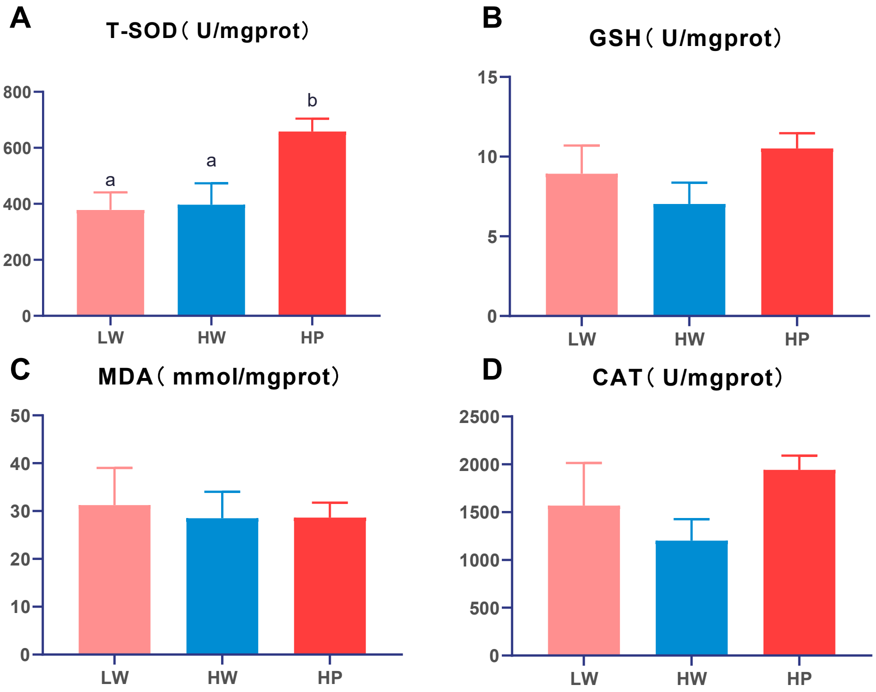

Figure S2. Effects of different experimental diets on liver antioxidant capacity of juvenile *M.* amblycephala. Note: The values in the figure are the mean ± standard error. The different lowercase letters indicated significant differences among groups (*P*<0.05, Turkey's Test).

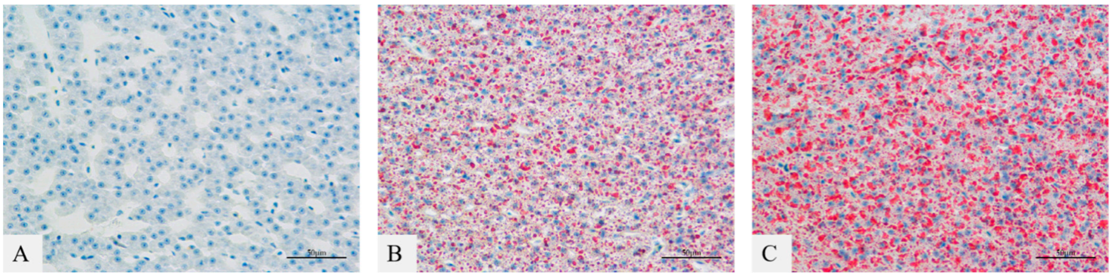

Figure S3. Histology analysis of hepatic lipid deposition in juvenile *M. amblycephala* using oil red O stainingNote: (A) LW group (×200), (B) HP group (×200), (C) HW group (×200). The red area presented lipid droplets, and the blue area indicated cell nuclei.

Table S1 Differential metabolites in LW and HW groups

Name	Formula	Molecular Weight	RT [min]	m/z	log2(HW/LW)	*P*–value	VIP
5−Methyltetrahydrofolic acid	C20 H25 N7 O6	459.1871	6.003	458.1802	–1.23	0.010515	2.0397
Taurochenodeoxycholic Acid (sodium salt)	C26 H45 N O6 S	499.291	13.943	498.2837	1.46	0.045888	2.3773
N1−{4-methyl-2-[(3,4,5-trimethoxybenzylidene)amino]phenyl}acetamide	C19 H22 N2 O4	342.1581	10.489	341.1511	1.02	0.034455	1.9353
LPI 20:3	C29 H51 O12 P	622.3122	14.194	621.305	1.21	0.011811	1.9747
Cucurbitacin I 2−O−β−D-glucopyranoside	C36 H52 O12	676.3449	7.655	675.3369	1.24	0.02745	2.7317
GlcADG (18:1-22:4)	C49 H82 O11	846.5695	15.19	845.5621	1.66	0.027409	3.1646
2'−O−Methyladenosine	C11 H15 N5 O4	281.1111	1.62	280.1037	–1.24	0.008719	2.4333
PC (16:1/20:5)	C44 H76 N O8 P	837.5523	14.95	836.5448	1.08	0.017164	2.388
PEtOH (20:3–20:4)	C45 H75 O8 P	774.521	16.195	773.5136	1.24	0.011538	3.9742
PEtOH (18:1–18:2)	C41 H75 O8 P	726.5211	16.346	725.514	1.29	0.039768	4.51
9(10) −DiHOME	C18 H34 O4	314.2457	13.217	313.2385	1.1	0.019905	2.1554
PG (18:1/18:2)	C42 H77 O10 P	772.5267	16.122	771.5198	1.39	0.017638	4.1072
PC (22:4/22:6)	C52 H84 N O8 P	941.6162	16.599	940.6088	1.19	0.026321	2.7498
PC (18:5e/18:2)	C44 H76 N O7 P	821.5597	15.65	820.5531	1.14	0.045665	2.7353
Milbemectin A4	C32 H46 O7	542.328	13.747	541.3207	–1.7	9.98E-07	3.4375
OxPC (16:0–22:5+1O(1Cyc))	C46 H80 N O9 P	881.5732	16.778	880.5667	2.72	0.011383	3.9533

4–amino–2–(4–chlorophenyl) –6–(methylthio)pyrimidine–5–carbonitrile	C12 H9 Cl N4 S	276.0289	1.361	275.0215	–1.39	0.013442	3.2449
S–Lactoylglutathione	C13 H21 N3 O8 S	379.1054	1.622	378.0975	–1.06	0.027949	1.9439
N–acetyl–D-–glucosamine	C8 H15 N O6	221.0901	1.542	256.0595	–1.04	0.019537	1.848
Cortisol	C21 H30 O5	362.209	11.143	363.2162	–1.55	0.003719	3.189
ACar 20:0	C27 H54 N O4	455.398	14.124	456.405	1.18	0.018463	3.1292
PC (18:5e/6:0)	C32 H56 N O7 P	597.3797	15.036	598.387	1.02	0.034627	2.6057
ACar 20:1	C27 H52 N O4	453.3817	13.954	454.389	1.23	0.007547	2.8927
N1-piperidinocarbonyl–4-–methylbenzene–1–sulfonamide	C13 H18 N2 O3 S	282.108	9.36	283.1153	–1.02	0.019448	2.0493
Dimetghyl 4–Hydroxyisophthalate	C10 H10 O5	210.0533	7.9	211.0605	–1.39	0.043802	2.2655
4–(3–methoxy–5,6–dihydrobenzo[c]acridin–7-–yl)morpholine	C22 H22 N2 O2	692.3288	8.037	693.3347	–1.51	0.002633	2.7781
ACar 24:4	C31 H54 N O4	243.1858	13.93	504.4054	1.27	0.026164	3.0851
Xanthurenic Acid	C10 H7 N O4	205.0379	7.099	206.045	–1	0.000102	2.2961
ACar 19:0	C26 H52 N O4	441.3818	13.997	442.3891	1.24	0.00169	3.0194
Caprolactam	C6 H11 N O	113.0842	0.383	114.0915	1.02	0.006732	3.0779
PC (16:2e/3:0)	C27 H52 N O7 P	533.3483	14.755	1067.701	1	0.041229	3.3075
Octyl hydrogen phthalate	C16 H22 O4	278.1514	13.419	279.1587	–1.93	0.014309	2.5156
LDGTS 22:6	C32 H51 N O6	545.3726	13.633	546.3799	–1.91	0.031831	4.137
ACar 17:0	C24 H48 N O4	413.3505	13.722	414.3575	1	0.009811	2.4412
Tetrahydrocortisone	C21 H32 O5	364.2248	11.067	365.2322	–1.85	0.001652	3.7624
Palmitoylcarnitine	C23 H45 N O4	399.3345	13.554	400.3418	1.12	0.035394	2.2589
ACar 15:0	C22 H44 N O4	385.3191	13.3	386.3264	1.04	0.035966	2.3822
JNJ–1661010	C19 H19 N5 O S	365.132	1.426	366.1392	–1.28	0.000451	3.1703
6αNaltrexol	C20 H25 N O4	343.1813	12.69	344.1885	–1.41	0.003614	2.8931

Table S2 Differential metabolites in LW and HP groups

Name	Formula	Molecular Weight	RT [min]	m/z	log2(HP/LW)	*P*–value	VIP
PG (16:1/16:1)	C38 H71 O10 P	718.4802	15.668	717.4729	2.5	0.034933	3.6428
PG (16:0/18:3)	C40 H73 O10 P	744.496	15.803	743.4882	2.14	0.009061	5.404
FAHFA (16:1/18:2)	C34 H60 O4	532.4502	14.27	531.4424	1.86	0.001957	3.3903
PC (22:6e/8:0)	C38 H66 N O7 P	679.4564	16.294	680.4634	1.81	0.002522	3.1508
Cucurbitacin I 2–O–β–D–glucopyranoside	C36 H52 O12	676.3449	7.655	675.3369	1.73	0.003092	3.3076
Lysopa 16:0	C19 H39 O7 P	410.2448	12.653	409.2375	1.72	0.002541	3.4909
N8–Acetylspermidine	C9 H21 N3 O	187.1687	1.163	188.176	1.72	0.02089	2.1203
PG (18:1/18:2)	C42 H77 O10 P	772.5267	16.122	771.5198	1.71	0.006168	4.1547
9(10) –DiHOME	C18 H34 O4	314.2457	13.217	313.2385	1.66	0.000809	2.9975
GlcADG (18:0–18:1)	C45 H82 O11	798.5704	15.061	797.5639	1.65	0.014615	3.4902
GlcADG (18:1–18:1)	C45 H80 O11	796.5539	14.881	795.547	1.48	0.018866	3.2565
PA (12:0/20:3)	C35 H63 O8 P	642.4208	14.56	641.4125	1.45	0.037384	1.7119
FAHFA (18:1/18:2)	C36 H64 O4	560.4776	14.663	559.4703	1.44	0.008707	2.6944
PEtOH (20:3–20:4)	C45 H75 O8 P	774.521	16.195	773.5136	1.43	0.0306	3.1696
20–Carboxy–Leukotriene B4	C20 H30 O6	366.206	8.093	755.4013	1.36	0.008791	2.3264
JWH 018 N–(4,5–epoxypentyl) analog	C24 H21 N O2	355.1542	7.778	356.1614	1.35	0.013068	2.2476
LPC 24:2	C32 H62 N O7 P	663.4481	16.415	662.4414	1.34	0.004359	2.2845
tetranor–12(R) –HETE	C16 H26 O3	266.1883	12.23	265.181	1.34	2.36E-05	2.4199
PC (18:5e/8:0)	C34 H60 N O7 P	625.4103	15.589	626.4179	1.32	0.016007	2.4965
Palmitoylcarnitine	C23 H45 N O4	399.3345	13.554	400.3418	1.31	0.034325	2.1226
LPC 24:1	C32 H64 N O7 P	665.4636	16.945	664.4564	1.29	0.005832	2.0376
PC (16:2e/8:0)	C32 H62 N O7 P	603.4271	16.212	604.4342	1.28	0.011217	2.3362
ACar 20:1	C27 H52 N O4	453.3817	13.954	454.389	1.27	0.003569	2.6935

FAHFA (16:1/18:3)	C34 H58 O4	530.4312	14.292	529.4241	1.2	5.12E-06	2.2355
Flurazepam–d10	C21 H23 Cl FN3 O	397.2158	1.508	398.2231	1.19	0.039966	2.3983
FAHFA (18:1/22:5)	C40 H66 O4	610.4937	14.677	609.4868	1.17	0.006412	2.2822
ADBICA N–pentanoic acid metabolite	C20 H27 N3 O4	373.1961	2.112	372.1888	1.17	0.000994	2.1764
Neopterin	C9 H11 N5 O4	253.0807	11.294	252.0734	1.15	0.001982	2.15
(+/-)12(13) –DiHOME	C18 H34 O4	314.2457	12.646	313.2385	1.14	0.000456	2.2738
FPH	C20 H25 N5 O4	421.1674	9.813	439.2007	1.13	0.02456	1.717
PC (17:2/22:6)	C47 H78 N O8 P	815.5351	15.396	816.5424	1.11	0.011944	2.5841
PMeOH (24:2–18:3)	C46 H81 O8 P	792.5624	14.871	791.5549	1.1	0.040234	2.4482
ACar 18:0	C25 H50 N O4	427.3659	13.873	428.3732	1.07	0.0304	2.0388
LPC 22:1	C30 H60 N O7 P	637.4324	16.248	636.4258	1.06	0.019201	2.0795
Natamycin	C33 H47 N O13	647.3039	6.724	648.3107	1.06	0.010943	1.9576
PC (22:6e/4:0)	C34 H58 N O7 P	623.3935	15.28	624.4014	1.05	0.034074	2.0886
Lysops 22:5	C28 H46 N O9 P	571.2891	5.988	570.2818	1.04	0.013079	2.0319
Arachidonoyl ethanolamide phosphate	C22 H38 N O5 P	427.2435	6.369	428.2508	1.02	0.035226	1.8368
23–Norcholic acid	C23 H38 O5	394.271	13.015	395.2783	1.02	0.026942	1.8151
3–Methoxy prostaglandin F1α	C21 H38 O6	368.25575	12.685	369.2629	1.12	0.018973	3.0146
N–P–Coumaroyl Spermidine	C16 H25 N3 O2	291.1946	3.419	292.2018	–1	0.036151	1.6212
Deoxyinosine	C10 H12 N4 O4	252.0861	5.608	253.0934	–1	0.009959	1.9127
D–Raffinose	C18 H32 O16	504.1694	1.621	503.1622	–1.01	0.002273	1.7316
Monobutyl phthalate	C12 H14 O4	222.0893	9.99	221.082	–1.02	0.027752	1.5284
LPS 20:1	C26 H50 N O9 P	551.3209	14.58	550.3136	–1.03	0.003044	2.1948
Thymine	C5 H6 N2 O2	109.0166	5.539	110.024	–1.03	0.03324	2.1605
5–Methyltetrahydrofolic acid	C20 H25 N7 O6	459.1871	6.003	458.1802	–1.04	0.04352	1.75
N1–(2,3-dihydro–1,4–benzodioxin–2–ylmethyl) –2,2–dimethylpropanamide	C14 H19 N O3	249.1398	10.435	250.1471	–1.04	0.026783	1.5497

2'–O–Methyladenosine	C11 H15 N5 O4	281.1111	1.62	280.1037	–1.05	0.022464	2.1316
8–Hydroxyquinoline	C9 H7 N O	145.0529	7.093	146.0601	–1.05	0.001813	1.9703
PC (14:0e/17:2)	C39 H76 N O7 P	684.501	14.738	685.5079	–1.05	0.000931	1.6909
L–(+)–Arabinose	C5 H10 O5	150.0531	1.622	299.0989	–1.07	0.020385	2.004
Methenolone	C20 H30 O2	302.2244	16.946	303.2316	–1.08	0.047312	1.359
N–lactoyl–phenylalanine	C12 H15 N O4	237.1002	9.591	238.1075	–1.09	0.000453	1.9102
PC (4:0/18:5)	C30 H50 N O8 P	601.3376	13.419	602.346	–1.1	0.036103	2.071
LPC 22:6	C30 H50 N O7 P	627.354	14.574	626.3469	–1.12	0.003451	2.4956
Fenpropimorph	C20 H33 N O	303.2558	11.362	304.2631	–1.19	0.000154	2.1965
1–Palmitoyl–Sn–Glycero–3–Phosphocholine	C24 H50 N O7 P	495.333	14.844	494.3254	–1.25	0.040475	1.7947
Dimetghyl 4–Hydroxyisophthalate	C10 H10 O5	210.0533	7.9	211.0605	–1.25	0.041662	2.25
Kynurenic acid	C10 H7 N O3	189.0431	7.112	190.0504	–1.29	3.62E-05	2.4313
Glycolithocholic acid	C26 H43 N O4	433.318	14.597	434.3257	–1.3	0.013105	2.3679
S–(Methyl)Glutathione	C11 H19 N3 O6 S	321.0987	2.36	322.1062	–1.3	0.003824	2.2622
S–Lactoylglutathione	C13 H21 N3 O8 S	379.1054	1.622	378.0975	–1.33	0.006116	2.9709
GPH	C13 H19 N5 O4	291.1315	1.515	292.1387	–1.35	0.019957	2.1553
Vitamin A	C20 H30 O	286.2295	14.909	287.2371	–1.35	0.024169	2.502
JNJ–1661010	C19 H19 N5 O S	365.132	1.426	366.1392	–1.4	1.32E-05	2.7436
Milbemectin A4	C32 H46 O7	542.328	13.747	541.3207	–1.47	0.000297	2.9102
Biotin	C10 H16 N2 O3 S	488.1756	8.491	489.1824	–1.47	0.005298	3.1601
Xanthurenic Acid	C10 H7 N O4	205.0379	7.099	206.045	–1.61	2.61E-06	3.1372
Folic acid	C19 H19 N7 O6	441.1376	8.848	442.1449	–1.93	0.031056	2.0896
PC (3:0/16:2)	C27 H50 N O8 P	547.3276	14.464	548.3351	–2.55	0.035405	2.701
αZearalanol	C18 H26 O5	344.1582	4.803	345.1654	–2.75	0.001159	3.674
2–Mercaptobenzothiazole	C7 H5 N S2	166.9865	10.448	167.9938	–4.66	5.23E-11	8.0038

Table S3 Differential metabolites in HW and HP groups

Name	Formula	Molecular Weight	RT [min]	m/z	log2(HP/HW)	*P*–value	VIP
Taurochenodeoxycholic Acid (sodium salt)	C26 H45 N O6 S	499.291	13.943	498.2837	–1.77	0.012894	2.7426
3–[4–(tert-butyl)anilino] –2–(3–thienylcarbonyl)acrylonitrile	C18 H18 N2 O S	310.1199	4.027	309.1126	1.17	0.00602	2.0906
Glutathione	C10 H17 N3 O6 S	307.084	7.738	306.0768	1.32	0.038281	2.1717
LPS 20:1	C26 H50 N O9 P	551.3209	14.58	550.3136	–1.22	0.00595	2.5912
PC (16:1/20:5)	C44 H76 N O8 P	837.5523	14.95	836.5448	–1.12	0.040901	2.7501
tert–butyl 4–{2–[(4–chlorobenzoyl)amino]ethyl}piperazine–1–carboxylate	C18 H26 Cl N3 O3	367.1652	11.963	366.1579	1.09	0.025015	2.1162
LPC 24:1	C32 H64 N O7 P	665.4636	16.945	664.4564	1.1	0.018334	1.7284
Ethyl–β–D-glucuronide	C8 H14 O7	222.0754	6.845	221.0681	2.09	0.02684	3.7706
2–Ketohexanoic acid	C6 H10 O3	130.0632	5.817	129.0559	1.29	0.016721	2.116
Corchorifatty acid F	C18 H32 O5	328.225	10.996	327.2177	1.29	0.036049	1.8625
Salvinorin B	C21 H26 O7	390.1714	9.984	389.164	1.86	0.042763	2.9989
LPC 20:5	C28 H48 N O7 P	601.3384	14.317	600.3312	–1.22	0.016161	2.8556
13,14–Dihydro–15–keto–tetranor prostaglandin F1α	C16 H28 O5	300.1937	9.876	299.1863	1.64	0.00566	2.7639
α–Ergocryptine	C32 H41 N5 O5	575.3177	11.959	574.3106	1.59	0.045968	4.1105
Lysopa 16:0	C19 H39 O7 P	410.2448	12.653	409.2375	1.02	0.04888	1.8246
Asp–Glu	C9 H14 N2 O7	262.08	1.35	261.0727	1.04	0.046737	1.7334
Neopterin	C9 H11 N5 O4	253.0807	11.294	252.0734	1.55	4.3765e-05	2.9505
LPC 22:6	C30 H50 N O7 P	627.354	14.574	626.3469	–1.25	0.004147	2.9277
Methionine sulfoxide	C5 H11 N O3 S	165.0461	1.351	166.0534	1	0.025327	1.581
Cortisone	C21 H28 O5	360.1935	11.2	361.201	1.32	0.028651	1.8163
L–Saccharopine	C11 H20 N2 O6	276.132	1.444	277.1391	1.09	0.037218	1.6244
PC (20:5e/20:1)	C48 H86 N O7 P	819.6142	14.773	820.6218	1.01	0.009179	2.2963
PC (3:0/16:2)	C27 H50 N O8 P	547.3276	14.464	548.3351	–2.97	0.024631	3.5483
αZearalanol	C18 H26 O5	344.1582	4.803	345.1654	–1.64	0.003766	2.6596

(1E) –5-hydroxy–1,7–diphenylhept–1–en–3–one	C19 H20 O2	302.1269	10.122	303.134	1.43	0.012475	2.1302
Albendazole sulfone	C12 H15 N3 O4 S	297.0823	5.35	298.0895	2	0.03639	2.4099
5–oxo–3–phenyl–5–(2–quinolinylamino)pentanoic acid	C20 H18 N2 O3	356.1095	1.271	357.1169	–1.07	0.022369	1.7714
PC (16:1e/15:0)	C39 H78 N O7 P	703.551	15.137	704.5584	2.27	0.023985	3.0493
Gelsemine	C20 H22 N2 O2	322.1668	7.283	323.1739	1.28	0.022695	1.8403
PC (18:5e/8:0)	C34 H60 N O7 P	625.4103	15.589	626.4179	1.06	0.048586	1.9334
PC (22:6e/8:0)	C38 H66 N O7 P	679.4564	16.294	680.4634	1.5	0.013621	2.665
GPH	C13 H19 N5 O4	291.1315	1.515	292.1387	–1.94	0.022595	2.8892
PC (18:5e/3:0)	C29 H50 N O7 P	555.3321	14.367	556.3394	–1.13	0.013775	2.2447
PC (4:0/18:5)	C30 H50 N O8 P	601.3376	13.419	602.346	–1.29	0.046076	2.4313
Prostaglandin A3	C20 H28 O4	332.2058	13.827	333.2131	1.8	0.002328	2.9633
2–Mercaptobenzothiazole	C7 H5 N S2	166.9865	10.448	167.9938	–4.1	1.4202e-08	7.4016

Table S4. Metabolites analyses with significant differences

Item (ng/μL)	LW	HW	HP	*P*-Value (Independent T test)			Function
				LW vs HW	HW vs HP	LW vs HP	
Xanthurenic Acid	6.97±0.04	6.58±0.07***	6.48±0.10^{+++}	***P*=0.001**	***P*=0.001**	*P*>0.050	Quinolines and derivatives
JNJ-1661010	7.32±0.03	6.81±0.03***	6.87±0.08^{+++}	***P*<0.001**	***P*<0.001**	*P*>0.050	Diazinanes
Neopterin	5.64±0.11$^{\#\#\#}$	5.57±0.06	6.01±0.08^{+}	*P*>0.050	***P*=0.018**	***P*=0.001**	Pteridines and derivatives
5-Methyltetrahydrofolic acid	6.02±0.13	5.66±0.02*	5.72±0.13	***P*=0.019**	*P*>0.050	*P*>0.050	Pteridines and derivatives
S-Lactoylglutathione	6.47±0.11	6.21±0.10	6.14±0.17	*P*>0.050	*P*>0.050	*P*>0.050	Carboxylic acids and derivatives
2'-O-Methyladenosine	7.72±0.06	7.50±0.07*	7.45±0.15	***P*=0.028**	*P*>0.050	*P*>0.050	Purine nucleosides
PG (18:1/18:2)	5.50±0.21	6.24±0.16*	6.55±0.13^{++}	***P*=0.019**	***P*=0.002**	*P*>0.050	Glycerophospholipids
PEtOH (20:3-20:4)	6.04±0.26	6.70±0.13*	6.73±0.16^{+}	***P*=0.046**	***P*=0.049**	*P*>0.050	Glycerophospholipids
PC (4:0/18:5)	7.15±0.11	7.24±0.07	6.92±0.16	*P*>0.050	*P*>0.050	*P*>0.050	Glycerophospholipids
PC (3:0/16:2)	6.45±0.25	6.46±0.24	6.07±0.09	*P*>0.050	*P*>0.050	*P*>0.050	Glycerophospholipids
PC (22:6e/8:0)	6.20±0.12	6.21±0.12	6.65±0.16	*P*>0.050	*P*>0.050	*P*>0.050	Glycerophospholipids
PC (18:5e/8:0)	6.21±0.16	6.37±0.13	6.60±0.15	*P*>0.050	*P*>0.050	*P*>0.050	Glycerophospholipids
PC (16:1/20:5)	7.53±0.08$^{\#}$	7.94±0.09**	7.60±0.09	***P*=0.007**	*P*>0.050	***P*=0.022**	Glycerophospholipids
Lysopa 16:0	5.68±0.16	6.00±0.09	6.19±0.13^{+}	*P*>0.050	***P*=0.033**	*P*>0.050	Glycerophospholipids
LPS 20:1	7.81±0.07$^{\#}$	7.95±0.10	7.50±0.13	*P*>0.050	*P*>0.050	***P*=0.024**	Glycerophospholipids
LPC 24:1	5.61±0.09	5.68±0.06	5.98±0.14^{+}	*P*>0.050	***P*=0.047**	*P*>0.050	Glycerophospholipids
LPC 22:6	8.57±0.07$^{\#}$	8.72±0.09	8.27±0.14	*P*>0.050	*P*>0.050	***P*=0.026**	Glycerophospholipids
ACar 20:1	7.79±0.14	8.11±0.12	8.09±0.10	*P*>0.050	*P*>0.050	*P*>0.050	Fatty Acyls
9(10)-DiHOME	6.60±0.07	6.83±0.13	6.97±0.13^{+}	*P*>0.050	***P*=0.030**	*P*>0.050	Fatty Acyls
Palmitoylcarnitine	8.67±0.12	8.88±0.14	8.89±0.18	*P*>0.050	*P*>0.050	*P*>0.050	Fatty Acyls
Taurochenodeoxycholic Acid	5.69±0.12$^{\#}$	6.17±0.18*	5.66±0.09	***P*=0.047**	*P*>0.050	***P*=0.030**	Bile acids, alcohols and derivatives
αZearalanol	6.11±0.17$^{\#}$	5.99±0.17	5.57±0.06^{+}	*P*>0.050	***P*=0.023**	***P*=0.039**	/
Milbemectin A4	7.44±0.06	7.02±0.03***	6.93±0.13^{++}	***P*<0.001**	***P*=0.006**	*P*>0.050	/

GPH	7.18±0.11	7.05±0.18	6.69±0.12^{+}	*P*>0.050	***P*=0.015**	*P*>0.050	/
Cucurbitacin I 2-O- β -D-glucopyranoside	5.40±0.15	5.90±0.15*	6.01±0.12^{++}	***P*=0.039**	***P*=0.010**	*P*>0.050	/
2-Mercaptobenzothiazole	7.04±0.11$^{###}$	6.84±0.13	5.64±0.04^{+++}	*P*>0.050	***P*<0.001**	***P*<0.001**	/

Note: An independent T- test was performed on the original data by $\log_{10}$. Data were shown as mean ± SEM. * indicated that LW group and HW group are significant and label it in HW group, $^{+}$ indicated that HW group and HP group are significant and label it in HP group, $^{\#}$ indicated that LW group and HP group are significant and label it in LW group. *$^{, +, \#}$ indicated P<0.05, $^{**, ++, \#\#}$ indicated P<0.01, $^{***, +++, \#\#\#}$ indicated P<0.001.
